# Dr. Jedediah Cobb

**Published:** 1861-01

**Authors:** 


### NECROLOGICAL NOTICES.

Dr. Jedediah Cobb.—With heartfelt sorrow we record the decease of this amiable, excellent, and distinguished physician, which took place, after a lingering illness, on the sixteenth of November, at Manchester, Massachusetts. He expired in the bosom of his loving and almost idolizing family, in the sixty-first year of his age. The immediate cause of his death was ulceration of the mucous membrane of the stomach. His health had, at times, from an early period of his life, been delicate, and many years ago he experienced occasional attacks of cecitis, from one of which he came very near perishing. When we last met with Dr. Cobb, six years ago, he looked hale, vigorous, and as if he might attain to a green old age. In August, 1858, his friends first began to notice that he was losing flesh and strength, and he himself soon became sensible that there was a marked decline in the state of his health. Indeed, from this time on, although there was occasionally a slight improvement in his condition, it was apparent to every one that there was a steady but gentle descent to the grave.

The nature of his disease remained for some time uncertain. Dr. Cobb himself was under the impression for several months that he was laboring under some pulmonary affection, but he finally concurred in the opinion of his medical attendants that the chief trouble was in the stomach. Although no post-mortem examination was made, this view of the case is strongly supported by the fact that he suffered greatly from nausea and headache, and that for a month before his death he was unable to retain any animal food. His emaciation also became excessive, but at no period of his illness was there any cough.

Until within a few weeks of his dissolution, his family had been making preparations to spend the winter with him in Florida, in the hope that a change of air and the balmy breezes of the South would at least prolong his life, if not restore his health. But his strength now so utterly failed him that they were soon compelled to abandon the project, and resign themselves to their fate. During all this time his cheerfulness and spirits never deserted him. He manifested to the last a lively interest in everything that passed around him, and thus deceived not a few of his friends in regard to the real state of his health. He had evidently set his house in order, and was calmly awaiting the message that was to summon him hence. His last hours were peaceful and happy, surrounded by his wife and children, who had loved him so long and so dotingly.

Dr. Cobb was born at Gray, in the State of Maine, on the 27th of February, 1800. His early education was obtained at Hebron Academy, and he entered Bowdoin College, at Brunswick, in September, 1816, graduating at the end of four years. Of his deportment while at these institutions, we have no knowledge; that it was eminently exemplary, no one can doubt; for as the character of the youth is, so is generally that of the man in the full development of his moral and intellectual faculties. Of his parentage we are also ignorant; but that the father and mother of Dr. Cobb were excellent people is an opinion amply sustained by the pure and lofty career throughout a long life of the son. Although we do not believe that bad parents must necessarily have bad or vicious children, yet it is unquestionably true that such a result is exceedingly common, if, indeed, it is not so general as to constitute a law.

During his residence at Bowdoin College, Dr. Cobb attended some of the medical lectures there; but he afterward went to Boston, where he became a private pupil of the late Dr. George C. Shattuck, an eminent and wealthy physician, who assisted in educating a number of the most prominent practitioners and teachers of New England. He took his medical degree at Harvard University, in September, 1823. He now went to Portland with a view of establishing himself in business, but he had been there only a few months when, through the influence of his late preceptor, for whom he always cherished a warm attachment, he was appointed Professor of the Theory and Practice of Medicine in the Medical College of Ohio, at Cincinnati, whither he accordingly removed during the following summer. His journey, as he often himself told us, was a long and tedious one, attended with much fatigue, and many little incidents calculated to make an enduring impression upon one so young and of so ardent and enthusiastic a temperament. When he reached the Ohio River at Pittsburg, no steamboat could be found small enough for the low stage of the water, and he was consequently obliged to take passage with several other gentlemen in a common flat-boat, a part of their duty consisting in rowing their little craft and cooking their own food, which was not always of the choicest quality. Many anecdotes were related by the merry crew, and after nearly a fortnight of hard work they finally arrived at the "Queen City," wearied and sunburnt, but in reality all the better for their protracted voyage.

His first course of lectures in the Medical College of Ohio was delivered in the winter of 1824–25, and the second the following year, when he was transferred to the chair of anatomy, which was much more in accordance with his tastes and previous studies. The fact is, he had accepted the chair of medicine with great reluctance, conscious that his youth and inexperience scarcely justified him in assuming its weighty responsibilities. Once in the harness, however, he shrunk from no duties, and there is reason to believe that he performed his task with more than ordinary credit; certainly with the most scrupulous fidelity.

He continued in the occupancy of the anatomical chair till his removal to Kentucky in 1837; and every one acquainted with the history of the Medical College of Ohio will admit that he contributed in no small degree, during his connection with that institution, to its influence and respectability. Prosperous it could not be said to have been at any time; for the demon of discord was always at work, in some way or other, impairing its usefulness, and often threatening its very existence. As a necessary result, there were frequent changes in its Faculties, not always, by any means, conducive either to strength or harmony. The truth is, the school was constantly in trouble, and it need, therefore, not excite any wonder that Dr. Cobb should have at length accepted the corresponding chair in the newly established college at Louisville, especially when he could thus readily exchange an income of a few hundred dollars, the net proceeds of his winter's toil, for three thousand dollars, so generously guaranteed to him for three successive years by the citizens of that noble town. He owed this call not to any extraneous influence, chance, or accident, but solely to his reputation as a teacher of anatomy, which had long ere this become as familiar as a household word with the profession in the great valley of the Mississippi.

Dr. Cobb entered upon his duties at Louisville with new zeal and vigor. Most of his colleagues had, like himself, already acquired extensive fame, and no doubt could, therefore, be entertained respecting the rapid and complete success of the school. Not less than four of his colleagues—Caldwell, Short, Cooke, and Yandell—had been approved teachers at Lexington; and Miller, since so widely known through his obstetrical writings, was already established in the confidence of the citizens of Louisville as an able and judicious practitioner. In 1838, Dr. Drake was added to the Faculty, which was now one of towering strength, "the observed of all observers" in the Southwest. Students flocked to the school from all sections of the valley of the Mississippi, and soon the classes attained an unparalleled size, vieing with those of Philadelphia and the most prominent colleges in foreign countries. Although we had known Dr. Cobb during his residence at Cincinnati, having occupied for a short time the position of Demonstrator of Anatomy in the Medical College of Ohio, by which we were thus brought into more or less close relations, our acquaintance with him only fairly commenced in 1840, when we became associated together as colleagues. A warm and intimate friendship soon sprung up, which happily continued until it was dissolved by his late lamented death.

In 1852, Dr. Cobb resigned his chair in the University of Louisville, and reentered the Medical College of Ohio, with an entirely new Faculty, of which Dr. Drake formed a conspicuous member. The chief motive which induced him to take this step, which all his friends regarded as an unfortunate one, especially in a pecuniary point of view, was that he might settle his eldest son, Dr. William Cobb, a youth of much promise, in the practice at Cincinnati, where he also held the position of Demonstrator of Anatomy, under the immediate superintendence of his father. The school, however, was again doomed to sad reverses. The session had hardly commenced before Dr. Drake, one of its main pillars, sickened and died; the class was small and not particularly remunerative; and toward spring, the health of Dr. Cobb failing, he considered it his duty to resign, bidding a final adieu to anatomical teaching and medical colleges. His son also abandoned Cincinnati, and settling in Missouri, died a few years after, thus casting a gloom over his father's spirits from which he did not soon recover. After his resignation, Dr. Cobb was offered the chair of anatomy in the University of Nashville, but for the reasons just mentioned was obliged to decline it.

Thus cut loose from pursuits which had so pleasantly and so profitably occupied him for nearly thirty years, during which he was brought into contact with some of the ablest medical men of the country, he looked about in search of a new home, and finally, in the spring of 1854, settled at Manchester, Massachusetts. He had long evinced an ardent desire to live near the ocean, to behold daily its dashing waves, and to be lulled to sleep at night under its wild and moaning sounds, and he had at length found the very spot which his heart had so long and so ardently coveted. Here, amid the wildest and most beautiful scenery, which might have charmed Calypso and her nymphs, in the presence of a glorious sky, he lived in profound retirement during the summer months, surrounded by his family, devoting his time to reading and the improvement of his small farm, with little or no intercourse with the profession, but with his house always open to his friends, whom he never failed to receive with all the cordiality of his warm heart. His winters were always spent at Louisville and Cincinnati, where he was sure to meet with warm, well-tried and sympathizing companions.

Dr. Cobb had a great aversion to writing, and hence he has left no enduring monument as an author. Indeed, we do not believe that all he has ever published would, if collected, make twenty pages. Even letter-writing, except on matters of a purely business character, was a great annoyance to him. His repugnance to authorship was invincible.

But if he was no writer, he was, nevertheless, a most useful teacher. As a lecturer on anatomy it is perfectly safe to say he never had a superior, and when we say this, we mean fully what we say. As a mere speaker he has, doubtless, often been excelled, but as a teacher, clear and lucid; as a demonstrator, thoroughly and intimately acquainted with his subject; as a dissector, wielding a ready and dexterous scalpel;—in all these qualities Cobb never was and probably never can be excelled. He was the very impersonation of a neat, gentlemanly, and finished lecturer. Slovenliness, in any form, he could not brook. The cadaver must always be fresh and sweet; the dissection exquisitely finished; the table so neat that one would hardly have hesitated to make his repast upon it. It was a pleasure to see him at his daily task, preparing for his twelve o'clock lecture. From two to three hours were usually spent in this way, most of the nicer labor being performed by himself; and one could not look at the result without being reminded of the expression of the Psalmist, "Man is fearfully and wonderfully made;" without feeling that every dissection of this excellent anatomist was in truth a sermon, or, to use the language of Galen, a hymn to the Deity.

Once fairly at work before his class, Cobb enchained the attention of every student; the hour passed rapidly by, and every one that listened to him felt how much he had been instructed. His popularity as a teacher was universal; and no man ever commanded greater respect than he did in the lecture-room; for he was a most admirable disciplinarian, never permitting the slightest noise or interruption during his demonstrations. No one was more keenly alive to everything of the kind than he was. The least whisper invariably disturbed him. His voice was sweet and sonorous, and sufficiently strong to fill the largest lecture-room.

In his intercourse with the world, his profession, and his colleagues his conduct was a model. He was eminently a man of principles; expediency he scorned. His manners were polished, elegant, and graceful, his language refined and well chosen, his presence and bearing those of the accomplished and high-toned gentleman. In person he was tall and rather slender, with a fine head, a black eye, shaded by a well-shaped brow, and a face at once beautiful—almost too beautiful for a man—and beaming with benignity. In short, he was a remarkably handsome man, one that would have attracted attention in any crowd of gentlemen. His heart was full of warmth, and as genial as a sunbeam. His social qualities were of a high order, and the consequence was that he was a general favorite with all classes of people. His moral character stood out in bold relief; he was a true man, a warm friend, and a generous enemy. To cherish resentment was no part of his nature.

Dr. Cobb never was fond of the practice of his profession; he disliked its details and its worrying and exhausting cares. Throughout life, in the winter, much of his time was occupied in the dissecting-room, and this circumstance, added to the fact that his health was for many years delicate, was probably the reason why he never had much business. Had he felt inclined to cultivate practice, he might have secured any amount of it, such was his personal popularity and the confidence reposed in his skill.

One of the remarkable traits of his character was his fondness for angling and shooting. Izaak Walton has had few more ardent disciples. The Little Miami, near Cincinnati, was, for many years, during every spring, a favorite place of resort with him, and during his summer wanderings to New England, fly-fishing was one of his most agreeable occupations. Few men could bring down a partridge or a woodcock with more unerring certainty than he.

In consequence of not being engaged in practice, Dr. Cobb acted for many years as Dean of the several Faculties of the schools with which he was connected, and his accuracy as an accountant was proverbial. As a colleague, his conduct was most exemplary. In 1830 he visited Europe, partly at the instance of the Trustees of the Medical College of Ohio, to make purchases for its museum and library. In 1836–37 he delivered two courses of lectures on anatomy at Bowdoin College.

In his domestic relations no man was ever more happy. The idol of his family, he warmly reciprocated the affection and tenderness so lavishingly bestowed upon him by his wife and children. He was married in 1826 to Ann Maria Merrill, who, with two sons and a daughter, all grown up, survives him.

In making a record of the death of one whom all loved, who had so many warm friends and no enemies, who shed a genial influence upon everything with which he came in contact, who discharged so well and so gracefully all the duties of official, social, and domestic life, we feel as if the world were the worse off for having lost so excellent a citizen, especially at a time when the Republic, groaning under the effects of political strife, is so much in need of true and good men. As we stand in imagination at his grave, we recall, with a melancholy satisfaction, his many virtues, and mingle our tears with those of his family.

